# Hepatitis Virus C-associated Nephropathy: A Review and Update

**DOI:** 10.7759/cureus.27322

**Published:** 2022-07-27

**Authors:** Elmukhtar Habas, Khalifa L Farfar, Nada Errayes, Ala M Habas, Mehdi Errayes, Gamal Alfitori, Amnna Rayani, Mohamed Elgara, Aisha H Al Adab, Abdulnaser Elzouki

**Affiliations:** 1 Internal Medicine, Hamad General Hospital, Doha, QAT; 2 Internal Medicine, Alwakra Hospital, Alwakra, QAT; 3 Epidemiology, University of Lincoln, Lincoln, GBR; 4 Internal Medicine, Tripoli Medical Hospital, Tripoli, LBY; 5 Hemat-Oncology Department, Pediatric Tripoli Hospital, Tripoli University, Tripoli, LBY; 6 Pulmonary Medicine, Hamad General Hospital, Doha, QAT

**Keywords:** hcv-associated gn, glomerulonephritis, gn, hcv kidney injury pathogenesis, cryoglobulinemia, hvc-associated nephropathy, hepatitis c

## Abstract

Hepatitis C virus (HCV) infection causes hepatic and extrahepatic organ involvement. Chronic kidney disease (CKD) is a prevalent non-communicable disorder, accounting for significant morbidity and mortality worldwide. Acute kidney injury and CKD are not uncommon sequels of acute or chronic HCV infection.

The pathogenesis of HCV-associated kidney injuries is not well explored. Excess cryoglobulin production occurs in HCV infection. The cryoglobulin may initiate immune complex-mediated vasculitis, inducing vascular thrombosis and inflammation due to cryoglobulin deposits.

Furthermore, direct damage to nephron parts also occurs in HCV patients. Other contributory causes such as hypertension, diabetes, and genetic polymorphism enhance the risk of kidney damage in HCV-infected individuals. Implementing CKD prevention, regular evaluation, and therapy may improve the HCV burden of kidney damage and its related outcomes.

Therefore, in this review, we discuss and update the possible mechanism(s) of kidney injury pathogenesis with HCV infection. We searched for related published articles in EMBASE, Google Scholar, Google, PubMed, and Scopus. We used various texts and phrases, including hepatitis virus and kidney, HCV and CKD, kidney pathology in viral hepatitis, kidney transplantation in HCV-infected patients, kidney allograft survival in viral hepatitis patients, mechanism of kidney pathology in viral hepatitis, dialysis and viral hepatitis, HCV infection and kidney injuries, and viral hepatitis and CKD progression, etc. to identify relevant articles.

## Introduction and background

Chronic kidney disease (CKD) is a prevalent non-communicable condition worldwide. Globally, CKD prevalence is about 10% [1], and in low-income nations, the prevalence is approximately 13.1% [2], equating to about 843.6 million cases worldwide in 2017 [3]. The global burden of CKD is rapidly growing, and it is projected to become the fifth most prevalent cause of death worldwide by 2040 [4]. Furthermore, the CKD burden increases faster in low- and middle-income countries [5]. CKD accounts for a significant fraction of the worldwide disease burden; however, over the past 27 years, the CKD burden has not decreased significantly as other non-communicable diseases. This increase is partially attributable to the higher prevalence of risk factors, such as hypertension (HTN), diabetes mellitus (DM), and obesity [6]. In the twenty-first century, CKD has become one of the primary causes of death and morbidity.

In 2022, the prevalence of hepatitis C virus (HCV) infection is estimated to be 56.8 million compared to 63.6 million in 2015. In 2017, the estimated prevalence of HCV infection was more than 180 million people, representing 2.8% of the global population [7,8]. Approximately 12.9 million (12.5-15.4) patients were estimated to have HCV infection at the end of 2020 [9]. HCV transmission is mainly by contacting blood or blood products. It has been reported that approximately 71 million individuals have chronic HCV infection globally, according to World Health Organization 2018 update in 2017 [10]. Worldwide, around 1.4 million hepatitis virus-infected patients die annually, which is more than the deaths caused by malaria or tuberculosis and nearly equal to the deaths caused by human immunodeficiency virus (HIV) [9]. The leading causes of death in chronic HCV infection are hepatocellular carcinoma and cirrhosis [11], which are increased by kidney involvement. The death rate is expected to increase during the next 10 years; for example, in England, it was reported that death would be higher in HCV-infected individuals than among the general population [12]. The increased number of deaths might be due to the increased number of undiagnosed chronic HCV cases [13], or unappreciated precautions of intravenous medications to treat other diseases [14].

HCV replicates in the liver cell, leading to fibrosis and eventually cirrhosis. Furthermore, HCV also replicates in B lymphocytes, activating cryoglobulin production. Moreover, the interaction between HCV and tissue proteins enhances the autophagy process [15] and the release of pro-inflammatory cytokines (interleukin(IL)-1, tumor necrosis factor (TNF), and IL-6) [15], promoting a chronic inflammatory response. The inflammatory response is linked with reactive oxygen species production [16] and nitric oxide formation [17], inducing kidney and liver damage due to unknown mechanisms [18].

CKD and HCV are epidemiologically related because (a) patients with CKD are more exposed to HCV in the dialysis units [19], and (b) HCV infection induces renal injury directly [20]. Furthermore, due to unknown mechanisms, HCV infection increases CKD risk and progression to end-stage renal disease (ESRD) [21,22].

HCV infection adversely affects the kidney graft and patient outcomes [23]. The therapy approach to HCV-infected patients has improved dramatically over the last few years. Substitution of interferon and ribavirin therapy by direct-acting antiviral agents, targeting precisely HCV proteins [24], has decreased the progression rate of extrahepatic complications [25], including kidney disease [20,26]. Despite the existing limitations about HCV access for screening and care, international guidelines advise all CKD patients to undergo laboratory HCV infection screen tests [27].

This review describes the pathogenetic mechanism(s) of HCV infection-associated kidney injuries. Achieving the aim of this review, the team agreed for phrases and texts to be used for searching PubMed, EMBASE, Google, Scopus, and Google Scholar for articles and reviews discussing this topic. The texts and phrases were hepatitis virus and kidney, HCV and CKD, kidney transplantation in HCV-infected patients, kidney pathology in viral hepatitis, kidney allograft survival in viral hepatitis patients, dialysis and viral hepatitis, mechanism of kidney pathology in viral hepatitis, HCV and kidney injuries, and viral hepatitis and CKD progression. The topic was divided into different sections, and then the sections were divided among authors to search and summarize the published article(s). The final manuscript was then handled and reproduced in its final form.

## Review

HCV infection prevalence in CKD, dialyzed, and kidney transplanted patients

In hemodialysis (HD)-dependent patients recruited for the first phase of the Dialysis Outcomes and Practice Patterns Study (DOPPS) during 2012-2015, the HCV prevalence was almost 10% [28]. HCV frequency in HD patients ranged from 4% in Belgium to 20% in the Middle East, with prevalence levels in China, Japan, Italy, Spain, and Russia being intermediate. HCV prevalence declined with time in most nations that participated in phase one of the DOPPS (14.3% decreased to 8.7%), and the prevalence among individuals who began HD just less than four months was approximately 5% [28].

Before applying mandatory HCV infection screening in dialysis and transplant divisions during the 1990s, HCV prevalence was greater because patients’ exposure risk for HCV was high. The highest prevalence was related mainly to nosocomial transmission between patients and the staff, HCV-contaminated blood transfusions, or infected allograft [29,30]. Improved hygiene, screening practices [19], and administration of erythropoiesis-stimulating agents have significantly decreased the risk of HCV infection among CKD patients in France [31]. Even with this marked reduction of HCV prevalence in CKD patients, its prevalence continues to be higher among patients with advanced CKD than in other populations [31,32]. Gookin et al. reported that the rate of death, anemia complications, worsening life quality scores, and increased hospitalization rate were higher in HCV-infected, HD-dependent patients [33].

The frequency of HCV infection in kidney transplanted patients declined by about 5% in the western world [33]. There is still some disagreement about the precise numbers of infected patients; however, a French registry study of people who began kidney replacement therapy (dialysis or a kidney transplant) between 2005 and 2013 observed that only 1.4% of them had HCV [31].

In a single-center retrospective study, CKD prevalence was more in HCV-infected patients compared to age-, race-, and gender-matched non-infected controls (p = 0.04) [34]. Another study revealed that the incident CKD risk was 27% greater in HCV-positive than in HCV-negative individuals [35]. Another report observed that the CKD risk of HCV-infected patients differed with age (8.5% at age 20-64 years versus 26.5% at age >65 years) [35]. A longitudinal cohort trial of 23,785 Taiwanese inhabitants found that the progression to ESRD was more in positive anti-HCV antibodies than in individuals with negative anti-HCV antibodies. Furthermore, HCV-associated GN is connected considerably to the viral load [26,36]. A Taiwanese study described that ERSD-associated death was higher between anti-HCV antibodies and HCV RNA-positive individuals than those with negative anti-HCV antibodies. Interestingly, the progression risk of ESRD was less in patients given interferon-based therapy than in untreated patients, suggesting its beneficial effects on improving kidney function [25].

Types of HCV-associated kidney pathology

**Figure 1:**
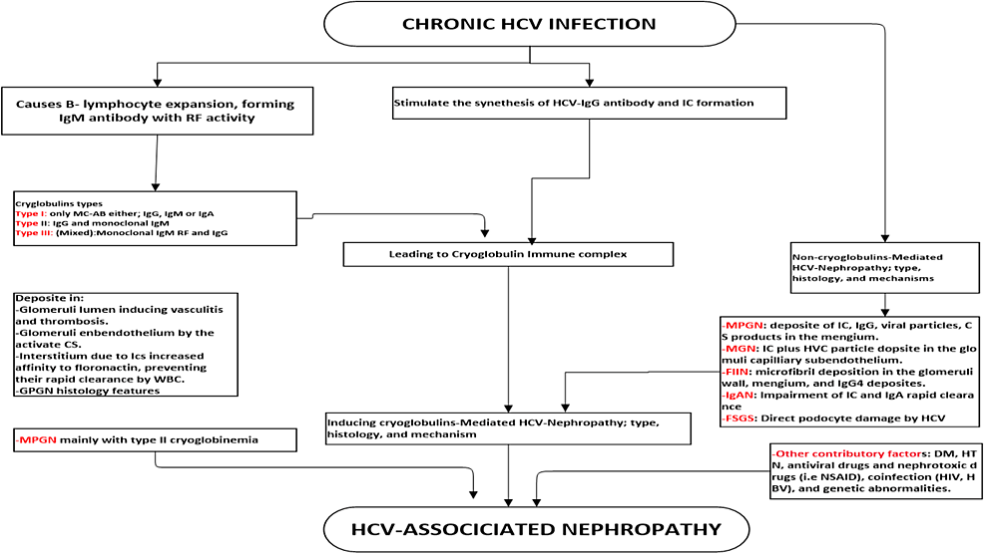
HCV-associated nephropathy: types and pathogenetic mechanism(s). RF: rheumatoid factor; CS: complement system; IC: immune complex; MPGN: membranoproliferative glomerulonephritis; MGN: membranous glomerulonephritis; IgAN: immunoglobulin A nephropathy; GS: glomerulosclerosis; FIIN: fibrillary immune-induced nephropathy; FSGS: focal segmental glomerulosclerosis; DM: diabetes mellitus; HTN: hypertension; MC-AB: monoclonal-antibody; HBV: hepatitis B virus; HIV: human immunodeficiency virus; NSAID: nonsteroid anti-inflammatory drug

HCV infection causes various histological injury patterns, impacting kidney function [37]. The frequently described lesions were membranoproliferative glomerulonephritis (MPGN) and membranous glomerulonephritis (MGN) [38]. Others uncommonly reported HCV-associated kidney lesions such as immunoglobulin nephropathy (IgAN), thrombotic microangiopathy, focal segmental nephropathy, fibrillary and immune-induced nephropathy, and medium- and small-vessel vasculitis, producing changes such as polyarteritis nodosa were observed [39]. Figure 1 summarizes the pathogenesis and the types of HCV-associated GN.

Cryoglobulinemic Nephropathy

Cryoglobulins are single or mixed immunoglobulins. They precipitate reversibly at low body temperatures (<37°C). Cryoglobulinemia can be idiopathic (primary) or secondary to systemic diseases. The clinical manifestations of cryoglobulins differ depending on their type [40]. A clinical syndrome of systemic inflammation caused by cryoglobulin-containing immune complexes precipitation may develop, affecting most frequently the liver, kidneys, and the skin. Cryoglobulins were categorized into three major types based on the circulating immunoglobulins [40]. Type I cryoglobulinemia is usually the product of monoclonal immunoglobulin (Ig) M, G, or A [41]. Type I cryoglobulinemia frequently occurs with plasma cell tumors. Mixed cryoglobulinemia occurs with autoimmune diseases and B-lymphocyte diseases such as non-Hodgkin lymphoma in 8-10% of the cases [42]. Type II and III cryoglobulinemia represent 80% of cryoglobulins. They contain rheumatoid factors, mostly IgM, and infrequently IgG or IgA.

Cryoglobulins precipitate in various organs, including the kidney, peripheral nerves, and joints. Furthermore, cryoglobulinemia is also divided into essential (idiopathic) and secondary subtypes based on the underlying disease. Cryoglobulinemia type I and type II have the exact prevalence (25% for each), while type III compromises 50% of the cases [43]. A link between HCV and mixed cryoglobulinemia was reported; however, it casts uncertainty on the presence of idiopathic cryoglobulinemia [44]. Secondary cryoglobulinemia is connected with specific conditions such as lymphoproliferative disorder, autoimmune disease, and infectious disease.

HCV is a lymphotropic virus that promotes lymphocyte differentiation to abnormal plasma cells. Type II and III cryoglobulinemia are highly related to HCV infection, and their involvement in HCV-associated kidney injury is recognizable [43]. Type II cryoglobulinemia is more significantly associated with HCV than type III (90% and 70%, respectively) [45]. The presence of cryoglobulins increases with the duration of the HCV infection. Notably, 6-28% of type II cryoglobulinemia patients develop overt lymphoma after 4-10 years of follow-up [46]. The production of mixed cryoglobulins is believed to be caused by partially uncontrolled B-cell clone expansion, frequently found in chronic HCV-infected individuals [46,47].

Type 1 cryoglobulinemia leads to renal involvement in almost 40% of cases, while two-thirds of patients have kidney lesions due to mixed cryoglobulinemia [48]. The most frequent kidney lesion associated with chronic HCV is MPGN [38,49,50] mainly due to cryoglobulinemia-induced vasculitis. HCV-induced MPGN is due to the precipitation of cryoglobulins in the glomerular endothelial layer cells, which is distinguished by fingerprint and subendothelial IgM and IgG deposition in the glomerulus capillary wall [51]. MPGN may occur without detectable cryoglobulins in some cases of HCV-infected patients, primarily due to an immune complex-mediated mechanism. In some patients with MPGN type I, subendothelial immune complex deposits without intraluminal precipitation have been observed [49].

All three types of cryoglobulinemia cause vasculitis, leading to kidney cryoglobulin-induced nephropathy. A study of 146 cryoglobulinemic patients with vasculitis‐associated renal lesions showed that 87% of patients were infected with HCV (98% genotypes 1b and 2). Approximately 85% of patients had MPGN, and about 7% had mesangioproliferative GN [47]. About 10-30% of chronic HCV-infected individuals develop MPGN type I.

The precipitation mechanisms of cryoglobulins are not perfectly understood. However, several aspects have been explored. It has been reported that light and heavy immunoglobulin chains contribute significantly to the solubility of cryoglobulins [49]. With a change in body temperature, the solubility of cryoglobulins varies, causing precipitate formation and vasculitis [52,53]. The ratio between circulating cryo-protein aggregates and immune complexes also significantly impacts the clearance and deposition of cryoglobulin complex [54]. Cryoglobulins may deposit in the vessel walls with C3a and C5a complement fragments. These deposits act as chemotactic mediators, inducing inflammatory processes. However, the mechanism is not entirely established [55].

Type II and III cryoglobulinemia are linked to chronic conditions such as rheumatoid arthritis, systemic lupus erythematosus, and chronic viral infections (e.g., chronic HCV). These conditions always have a polyclonal IgG fraction accompanied by monoclonal (type II) or polyclonal (type III) IgM (rarely IgA or IgG) with rheumatoid factor activity, which has a high affinity to attach with IgG. In these conditions, clonal growth of B-cells, particularly RF-secreting cells, is a distinguishing characteristic [41], enhancing the associated kidney injuries.

The deposition of immune complexes may lead to inflammatory vasculitis, aggravating the nonobstructive injury. In vivo, cryoglobulin precipitation may lead to small arteries and capillaries thrombus formation, causing glomeruli damage and acute kidney injury. Additionally, due to the high molecular weight of cryoglobulins, hyperviscosity syndrome develops, increasing the risk of thrombosis if they are not precipitate. The resulting aggregates and immunological complexes have been thought to exceed reticuloendothelial-clearing activity, increasing their deposit rate in the tissues. Notably, HCV-related particles are expected to be directly involved in HCV-associated kidney disease development because these particles are frequently present in the blood vessels of the kidney [51,56]. Pocino et al. reported that IgG subclasses, radio-frequency, and free light chains are potential biomarkers for the early detection of HBV-associated cryoglobulinemia [57].

MGN

The lack of significant cellular proliferation, increased glomerular basement membrane (GBM) thickness, and increased subepithelial immune deposits are distinctive features of MPGN. Two arguments encourage the presence of a direct causative correlation between MGN and chronic HCV infection. First, in an Egyptian series of patients, although almost all had MPGN lesions, 4% of patients had an apparent lesion of MGN only without any features of cryoglobulinemia [50]. Second, some patients detected HCV-related proteins in the glomeruli capillary wall [58], with minimal or no cryoglobulins precipitates.

MGN occurs in HCV-infected patients and can present with histological and clinical features similar to primary MGN [38]. According to Yamabe et al., 8.3% of patients with MGN had anti-HCV antibodies or detectable HCV RNA [59]. HCV-infected people are developing MGN due to immune complexes containing particles such as HCV proteins that accumulate in the glomeruli, which are detectable by electron microscopy [58]. This theory is significantly supported by the glomerular deposition of IgM, IgG, IgA, and complement products with HCV particles in HCV patients with MGN lesions [60].

Focal Segmental Glomerular Sclerosis (FSGN)

FSGN is an infrequent GN in HCV-infected patients. Patients may exhibit symptoms of primary FSGS, which makes it challenging to make the diagnosis; however, HCV infection laboratory and clinic features help differentiate between the two types. The fundamental pathogenic mechanisms are unknown; however, HCV is suspected to damage podocytes directly, just like HIV-associated kidney injuries, causing segmental glomerulosclerosis [61].

Other Rare HCV-associated Glomerulopathies

There are few case reports of fibrillary immune-mediated glomerulopathy in HCV-infected individuals. Extracellular mesangial microfibril deposits were observed, and glomerular capillary wall deposits were stained positively for IgG4 and C3 [62] but not negatively for Congo red staining [36]. Fibrillary immune-mediated glomerulopathy may present with renal failure, blood and protein in the urine, and HTN manifestations mainly due to nephritis [63].

HCV infection can cause IgAN-like lesion [64]. Serum IgA level increases in cirrhotic patients because the liver clears IgA and IgA-containing immune complexes, increasing the chance of IgA deposition into the nephron glomeruli. IFN administration to HCV-induced IgAN patients induces remission, which can support the pathogenic association between the two disorders [65]. However, it appears that HCV is not the only condition that connects to the development of IgAN in cirrhotic patients [66].

Pathogenesis of kidney involvement in HCV infection

HCV injuries the kidneys by two possible mechanisms: (a) immune-mediated mechanism, including effects from HCV lymphotrophism and cryoglobulinemia, and (b) direct virus attacks on nephron tissue (Figure 1).

Immune-Mediated Kidney Lesions in HCV

HCV antigens may activate B lymphocytes to produce autoantibodies, immune-mediated cryoprecipitates, and non-cryoprecipitate complexes [38,67]. The formed immune complex contains HCV or its remnants deposit in the nephron tissue, causing glomerular inflammation, initiating anti-HCV-IgG, complement activation, and anti-endothelial antibody production. This local inflammatory process provokes fibrinoid necrosis of glomeruli capillaries. This process causes adhesion molecule overexpression, recruiting different cell types, such as dendritic cells, natural killer cells, T and B cells lymphocytes, and dendritic cells, activating the thrombocyte and promoting its aggregation. Severe GBM damage allows the cryoglobulins to filter into the nephron tubules, forming glomeruli crescentic lesions and tubular casts [38,68,69]. Furthermore, Fabrizi et al. observed that MPGN type I typically is caused by type II mixed cryoglobulinemia in HCV infection. The same study found a correlation between HCV infection and proteinuria, suggesting that HCV-associated kidney disorders may be more prevalent than previously believed [69].

Direct Effects of HCV on the Kidneys

Besides the indirect effects of HCV infection on the kidney via the formation of immune complexes, it is tempting to speculate that the virus can directly affect and damage the nephron, negatively altering kidney function parameters. HCV can promote endothelium and tubules epithelial cell damage and enhance the migration of leukocytes and infiltration of the insulted tissues [50].

Kidney tissue may express CD81 [70], which is believed to be the receptor through which HCV infects B lymphocytes and liver cells [71]. HCV-infected endothelium cells may undergo apoptosis similar to blood-brain barrier endothelial cells damaged by radiation [72], causing more endothelial damage to nephron glomeruli and tubules. Furthermore, HCV damages Bowman capsule epithelial cells and causes podocyte injury [51].

Other Contributory Conditions Promote HCV-associated Nephropathy

The coexistence of HTN, DM, some genetic abnormalities [73], and nephrotoxic agents [74] increase renal failure risk in HCV-infected persons. HCV-positive patients have a 2-2.5-fold increase in experience of cardiovascular, cerebrovascular, or renovascular problems [74-76]. Additionally, HCV-infected persons are 1.5 times more likely to develop insulin resistance and type 2 DM than HCV-negative individuals [68,73,77]. HTN and DM have known cofactors, which could increase CKD and HCV-associated kidney complications, as well as accelerate CKD onset and progression to ESRD.

Interestingly, some reports support gene polymorphisms as a factor in increasing cryoglobulinemia production in HCV-infected persons. A study of 803 patients with positive HCV RNA described a significant association between chromosome 6 abnormalities, major histocompatibility complex (MHC) class II, Notch receptor 4 (NOTCH4) genes, and cryoglobulinemia-induced vasculitis. However, the specific gene accountable to the association is not recognized. Additionally, a polymorphism of the gene determined as IL-28B (also named IFNλ3) correlates well with spontaneous HCV infection clearance positive response to interferon and ribavirin therapy. This gene polymorphism is connected to mixed cryoglobulinemia following HCV infection [78]. A case-control study of 342 individuals illustrated a relationship between gene polymorphisms of chemokine receptor CCR5 and the inflammasome component NLRP3 and HCV-related kidney damage onset [79]. Therefore, follow-up is vital for HCV-positive patients receiving any drug that may induce kidney damage, including some anti-HCV agents [80].

Impact of HCV infection on renal graft

The death rate in dialyzed HCV-positive subjects is partly a result of the prevalence of cardiovascular and DM [68,81], besides the chronic HCV-related hepatic complications such as cirrhosis and hepatocellular carcinoma [26,82-84]. Reduced survival duration and early graft loss in HCV-infected kidney transplant individuals compared to non-infected HCV individuals are increased, mainly due to other contributing diseases such as cirrhosis-associated sepsis, chronic liver disease complications, and the adverse effects of immunosuppressive therapy [23,85,86]. In a Swedish study of 545 kidney transplant and 26 combined kidney and pancreas transplant patients, HCV infection significantly affected graft function and patient death than the dialysis duration [23]. An analysis of 23,046 patients on dialysis from the Australian and New Zealand Dialysis and Transplant registry found that those who had anti-HCV antibodies (1.6% of patients) had a higher mortality rate than those who had negative anti-HCV antibodies (adjusted hazard ratio = 1.25, 95% confidence interval = 1.07-1.46) [85]. An analysis of 7,572 renal transplant recipients from the same registry noted that recipients with serum-positive anti-HCV antibodies had a poor prognosis and more graft loss rate than negative HCV antibody recipients [85].

The associated hepatic and extrahepatic complications due to HCV infection are associated with increased morbidity and death rates in kidney transplant recipients [87]. HCV antibodies are independent predictors for patient survival and graft malfunctioning rate after a 10-year follow-up of HCV-positive renal transplant individuals [53]. There is no conclusive proof that anti-rejection treatment type and HCV activity in kidney transplant recipients are related. A study suggested that mTOR inhibitors may help reduce viral replication [88]. A balance between decreasing the viral load, drug side effects, and the increased risk of acute rejections should be considered [89].

HCV patients experience acute rejection, de novo or recurrent glomerular disorders, and accelerated kidney graft fibrosis [90]. MPGN develops in 5-54% of kidney transplant HCV-infected patients [91]. Positive anti-HCV antibodies in patients planned for renal transplantation are independent factors for proteinuria development later and lower graft survival rates [92]. Compared to HCV infection alone, coinfection with HIV is an additive risk factor for graft failure and patient death [93]. As recently expressed by Rallón et al., immunological deficiencies caused by HCV speed up the progression of HIV illness; therefore, early anti-HCV treatment in HIV coinfection is advisable [94].

Recommendations for HCV screening

HCV-infected patients are usually symptomless or may present with minimal symptoms and signs. Therefore, regular checking for anti-HCV antibodies is essential to identify HCV infection, especially in CKD, dialysis-dependent, and kidney transplant patients. Furthermore, KDIGO guidelines recommend HCV infection screening for patients before initiating HD and those who will be transferred to another HD center [27]. Additionally, patients on dialysis who have negative HCV serology should be regularly screened at least every six months.

Prompt reporting of any new HCV infection in a dialyzed patient is mandatory to the relevant public health authority. Furthermore, HCV antibodies and antigen testing for every patient in that center are obligatory. Serum alanine aminotransferase (ALT) monitoring is not expensive and is a good screening test for acute liver insult. Therefore, serum ALT levels must be determined for every CKD patient regularly, especially HD-dependent patients, because a minimal unexplained increase in serum ALT might indicate a new HCV infection.

## Conclusions

A collaborative, multidisciplinary approach between kidney and hepatology specialties is mandatory to prevent the complications of HCV and provide appropriate approaches.

The grand challenges are preventing and eliminating HCV in CKD HCV-positive, dialyzed, and transplanted patients. Furthermore, easy access to direct-acting antivirals and regular follow-up are other challenges to health authorities. The approaches should focus on patient and staff education about infection transmission, improving access to direct-acting antivirals, follow-up, and HCV transmission prevention among patients and staff in the units.

The evidence suggests that the currently utilized agents can be used in CKD patients, including those on maintenance dialysis or recipients of kidney transplants; however, their adverse effects on the kidneys must be considered. Studies have demonstrated that achieving a sustained virologic response for 12 weeks or more definitely affects the manifestations of HCV infection. However, more research is needed to assess HCV-associated kidney injuries, their possible pathogenesis mechanisms, new therapies, and how to prevent the hepatic and extrahepatic complications of HCV.
